# *MMWR* publications during the lapse in government funding

**Published:** 2013-10-11

Because of the current lapse in government funding, *MMWR* is able to publish and distribute only reports on immediate health threats.

